# Novel equine tissue miRNAs and breed-related miRNA expressed in serum

**DOI:** 10.1186/s12864-016-3168-2

**Published:** 2016-10-26

**Authors:** Alicja Pacholewska, Núria Mach, Xavier Mata, Anne Vaiman, Laurent Schibler, Eric Barrey, Vincent Gerber

**Affiliations:** 1 Department of Clinical Veterinary Medicine, Swiss Institute of Equine Medicine, Vetsuisse Faculty, University of Bern, and Agroscope, Länggassstrasse 124, 3012 Bern, Switzerland; 2 Department of Clinical Research and Veterinary Public Health, Institute of Genetics, Vetsuisse Faculty, University of Bern, Bremgartenstrasse 109A, 3012 Bern, Switzerland; 3 Animal Genetics and Integrative Biology unit (GABI), INRA, AgroParis Tech, University of Paris-Saclay, 78350 Jouy-en-Josas, France

**Keywords:** Horse, miRNA, microRNA, miRNome, Serum, sRNA-seq

## Abstract

**Background:**

MiRNAs regulate multiple genes at the post-transcriptional level and therefore play an important role in many biological processes. It has been suggested that miRNA exported outside the cells contribute to inter-cellular communication. Consequently, circulating miRNAs are of particular interest and are promising biomarkers for many diseases. The number of miRNAs annotated in the horse genome is much lower compared to model organisms like human and mouse. We therefore aimed to identify novel equine miRNAs for tissue types and breed in serum.

**Results:**

We analysed 71 small RNA-seq libraries derived from nine tissues (*gluteus medius*, *platysma*, *masseter* muscle, heart, liver, cartilage, bone, total blood and serum) using miRDeep2 and miRdentify tools. Known miRNAs represented between 2.3 and 62.9 % of the reads in 71 libraries. A total of 683 novel miRNAs were identified. Breed and tissue type affected the number of miRNAs detected and interestingly, affected its average intensity. A total of 50 miRNAs in serum proved to be potential biomarkers to differentiate specific breed types, of which miR-122, miR-200, miR-483 were over-expressed and miR-328 was under-expressed in ponies compared to Warmbloods. The different miRNAs profiles, as well as the differences in their expression levels provide a foundation for more hypotheses based on the novel miRNAs discovered.

**Conclusions:**

We identified 683 novel equine miRNAs expressed in seven solid tissues, blood and serum. Additionally, our approach evidenced that such data supported identification of specific miRNAs as markers of functions related to breeds or disease tissues.

**Electronic supplementary material:**

The online version of this article (doi:10.1186/s12864-016-3168-2) contains supplementary material, which is available to authorized users.

## Background

MiRNAs are endogenous ~22-nt noncoding RNAs that impact molecular mechanisms through the regulation of mRNA post-transcription and/or translation [1]. Regulation of gene expression by miRNAs requires direct interaction between the mature miRNA and, most often, the 3’ untranslated region of target mRNAs. Changes in the expression pattern of a miRNA can expose novel sets of genes to its regulatory influence [2]. The great current interest in miRNAs reflects their implication in diverse developmental processes and in the pathogenesis of cancer, cardiac, immune-related, pulmonary and other diseases [3]. Moreover, they are more stable, even outside a cell, compared to other long RNA molecules as they are either packed into microparticles, or protected by associated RNA-binding proteins (Argonaute 2) or lipoprotein complexes, which protect them from the RNAses activity [4]. Therefore, circulating miRNAs can be present in serum and other body fluids during the pathogenesis of many disorders [1]. Circulating and whole blood miRNAs (which contains cells that circulate throughout the body) profiles are thus of particular interest as promising non-invasive disease biomarkers [3, 5–8].

Over 700 miRNAs have been identified in the equine genome with distinct subsets of miRNAs differentially expressed in a tissue-specific manner [9, 10]. For example, horse specific miRNA in cartilage and bone have been already reported [10] and the existence of hundreds of horse specific transcripts may suggest the existence of more horse specific miRNAs [11]. Due to their strong sequence conservation, a majority of equine mature miRNAs have been perfectly matched to human miRNAs [4, 12]. The fact that many human miRNAs have been associated with human diseases indicates the potential of investigating miRNA profiles in equine pathologic conditions. However, the number of miRNAs studied and annotated in the equine genome is still much lower (*n* = 771) compared to human (*n* = 2,813) or mouse (*n* = 2,045) genomes, according to miRBase v.21 [13]. Moreover, some miRNA may be expressed in a breed-specific manner [14].

Current technology, i.e. small RNA sequencing (sRNA-seq), is able to provide a comprehensive study to determine the precise extend of miRNA profiles in horse. Taking into account the specific biogenesis of miRNA it is possible to distinguish short sequences that may be derived from degraded mRNA and not from true miRNA sequences [15]. The secondary structure of pre-mature miRNA resembles a hairpin, which is then processed by the endonuclease Dicer to form miRNA duplexes. MiRNAs in either the 5’ or 3’ end of their precursors are loaded into an RNA-induced silencing complex, which yields functional miRNAs. The loop and star sequences are usually degraded and therefore the highest coverage of the reads is expected within the mature miRNA region [15].

In this study we used combined high-throughput sequencing and bioinformatics methods to identify equine genome miRNAs. MiRNA profiles were thoroughly analysed by combining small RNA sequencing for multiple tissues to search for novel equine circulating and tissue-specific miRNAs. We prepared a total of 71 sRNA-seq libraries from six solid tissues, blood and serum from equine samples. Two different algorithms were applied to support the authenticity of the identified miRNAs: miRDeep2 [16] and the more stringent, miRdentify [17]. Given the miRNA’s fundamental role in transcriptional and translational regulation in different pathologies, we compared the expression profile of miRNA derived from *gluteus medius* (GM) muscle of healthy horses and horses affected with polysaccharide storage myopathy type I (PSSM). We also hypothesized that the identification of differences in the serum miRNA expression profile between Warmbloods and ponies could reveal unique biomarkers of equine breeds.

## Results

### Small RNA sequencing and mapping

We obtained small RNAs reads derived from 49 (HiSeq) and 22 (SOLiD) libraries. SOLiD samples were prepared from *platysma*, *masseter* and *gluteus medius* muscles, heart, liver, cartilage, bone and blood of 35 horses. MiRNA from the *platysma* and *masseter* muscles, heart and liver were extracted from 15 healthy horses of different ages and breeds (collected at the slaughterhouse). The HiSeq samples included serum RNA samples from 44 Warmbloods horses and five ponies (four Shetland and one Welsh pony). More details are given in Additional file 1: Table S1.

After quality filtering for low quality reads and trimming for adaptors sequences (see Methods for details), HiSeq data from serum consisted of a total of 12 M reads on average with mean per sequence quality of 38 in the Phred scale [18] per library. In these libraries, the size distribution followed the RNA length distribution reported in other equine and bovine serum samples [19, 20], with the majority of the reads (78 %) falling into piwi-interacting RNA region (30–32 nt) and with a smaller peak (4 %) in the mature miRNA region (22–23 nt). On the other hand, after removal of low-quality reads, the flanking linker and primer sequences, SOLiD data comprised 8 M reads on average with mean per sequence quality of 27 in the Phred scale per library. The reads longer than 30 nt were filtered out to exclude short fragments potentially derived from degraded cellular mRNA (see Methods) and finally the majority of the reads (77 %) were 21–23 nt long.

**Fig. 1:**
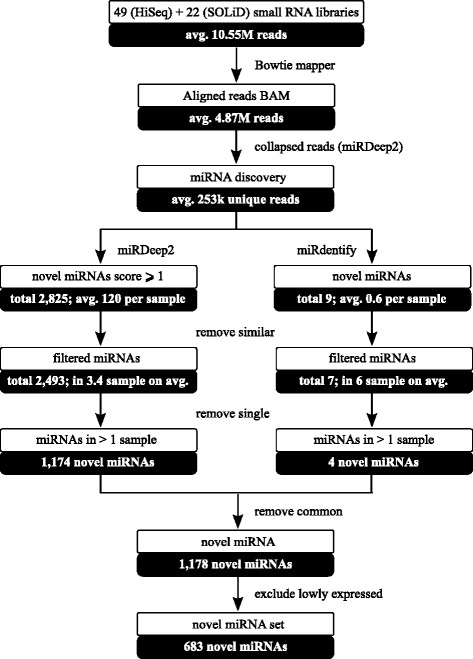
Workflow of the miRNA discovery analysis. The steps taken for the identification of 683 novel equine miRNAs from a total of 71 sRNA-seq libraries

**Fig. 2:**
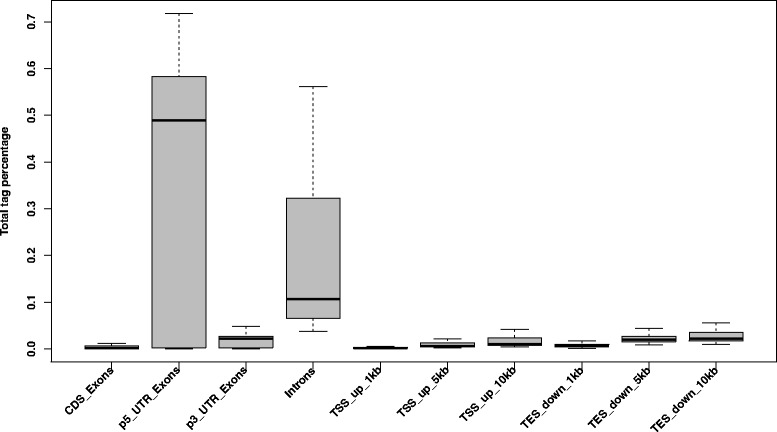
Distribution of reads mapped within 10 kb up- and downstream of the gene coding regions. The percentage of reads per library mapped to coding DNA sequences (CDS), 5’ and 3’ untranslated regions (UTR), introns, and within 1, 5, and 10 kb upstream of the transcription start site (TSS) and downstream of the transcription end site regions of the coding genes is shown across all samples. Known miRNA genes were excluded from this analysis

All remaining small RNAs from HiSeq and SOLiD data (748 M sequencing reads; 11 M on average per library) were aligned to the *Equus caballus* reference genome (EquCab2; v2.74) with BowTie [21]. Overall, 91 % of reads were mapped, but 336 M (45 %) were filtered out due to multiple mapping (more than 5 hits). After collapsing reads that had identical sequence (346 M tags; 46 %), 253,000 unique sequences were obtained per library on average (Fig. 1). Only 0.39 % of reads mapped to exonic regions of the protein-coding genes (coding DNA sequences; CDSs), 38 % mapped to 5’ untranslated regions (UTR), and 18 % mapped to introns (Fig. 2). The fraction of reads mapped to repeat elements, rRNAs, tRNAs, other noncoding RNA and repeated-related regions ranged from 12.7–93.4 %.

From 2.3 to 62.9 % (18.8 % on average) sequencing reads per library mapped to the known miRNA genes in agreement with the miRNA biogenesis [15]: most reads mapped mature -3p and -5p sequences of the miRNAs (Additional file 2: Figure S1).

### Novel equine miRNA discovery

**Fig. 3:**
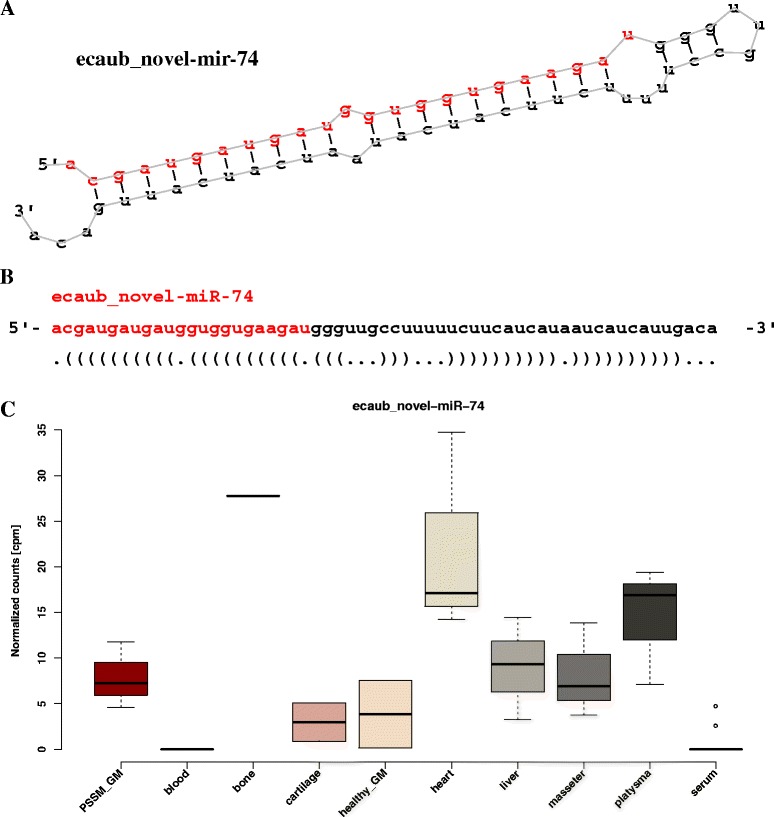
An example of novel miRNA structure and expression across tissues. The secondary structure of ecaub_novel-mir-74 predicted with RNAfold in a graphical (**a**) and Vienna format (**b**). The mature miRNA sequence is indicated in red. Boxplots represent the expression levels of ecaub_novel-miR-74 across the 10 tissues studied (**c**)

Two complementary algorithms were used to identify miRNAs in equine tissues: i) miRDeep2; and ii) miRdentify. Briefly, we used equine known mature and hairpin structure and human mature sequences as guidance (miRBase v. 2); and minimum miRDeep score of 1 (−t Horse -b 1). Among 781 known equine miRNAs expressed in our dataset (10 more mature and hairpin combinations identified by mirDeep2 compared to miRBase), from 131 to 256 were expressed per library (average = 183 (27 %)). The number of known miRNAs per library identified by miRdentify ranged from 14 to 164 (average = 56). A total of 2,493 regions in the equine genome expressed in the dataset met the miRDeep2 criteria and thus were considered as novel candidate miRNAs by miRDeep2, whereas only seven met the criteria of miRdentify. More than half of the novel miRNAs predicted by miRDeep2 (*n* = 1,319; 53 %) and three miRNAs predicted by miRdentify were identified only in a single library and were thus filtered out (Fig. 1). The remaining 1,174 novel miRNAs were scored from 1 to 30,339 (mean = 35.6). The four novel miRNAs identified by miRdentify partially overlapped with known miRNAs: the ecaub_novel-miR-1175 was only two nucleotides shorter than eca-mir-744, ecaub_novel-mir-1176 overlapped the position of an Ensembl predicted *ENSECAG00000025869*, whereas the ecaub_novel-mir-1177 was identified on the opposite strand of the eca-mir-486, and ecaub_novel-mir-1778 was located in the region of another Ensembl predicted *ENSECAG00000026103*. None of the final set of the novel miRNAs identified by miRdentify was simultaneously identified by miRDeep2. The range of novel mature sequences length was 19–25 nt with an average and median at 22 nt. An example of a novel miRNA secondary structure with expression across tissues is shown in Fig. 3.

When novel miRNAs were added to known miRNA set, the number of clustered miRNA (defined as at least 2 miRNAs within 3000 distance [22]) increased from 53 to 86, from which 15 could be identified among novel miRNAs separately.

### Equine miRNA expression

**Fig. 4:**
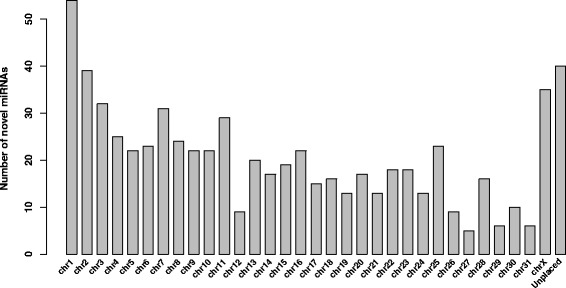
Novel miRNA distribution across chromosomes. The number of novel miRNA identified per chromosome. Unplaced scaffolds are shown as one chromosome “Unplaced”

For the known and discovered putative miRNAs, we investigated their expression levels in the small RNA libraries. A total of 2,093 unique combination of mature and hairpin sequences were expressed in our dataset (one mature miRNA may derive from more than one hairpin, also one or two mature miRNA derive from a single hairpin structure [4]). The mean expression of trimmed means of M values (TMM) of known (miRBase, release 21) and novel miRNAs ranged from 0 to 140,700 count per million (cpm), with an average of 0.64 cpm per miRNA. We then removed lowly expressed miRNAs (<1 cpm in 90 % samples), as they seem to play minor roles in gene expression regulation [23–25]. We detected 965 distinct mature miRNAs (280 known and 685 novel) expressed from 951 unique hairpin sequences (268 known and 683 novel) by combining the results from the two approaches. In total, there were 1,080 unique combinations (mature miRNA expressed from hairpin) with variable expression levels (0.20 to 140,700 cpm; median = 1.96 cpm) in at least one small RNA library. Among the 965 mature miRNAs, we observed that miR-486 was the most abundant, together with a putative novel miRNA identified in the opposite strand of miR-486, which was given here an identifier name ecaub_miR_1177. These two miRNAs were highly expressed in serum and blood samples compared to other tissues (false discovery rate (FDR) = 1.05e-59 and 3.72e-60, respectively; Additional file 3: Table S2). The highest number of novel miRNAs was identified on chromosome 1 (*n* = 54) and only five novel miRNA were identified on chromosome 27 (Fig. 4). Among the 683 novel miRNAs, 334 (50 %) were transcribed from the sense (+) strand of the DNA (Additional file 4: Table S3). After removal of the lowly expressed miRNAs, the length of the final set of novel mature miRNAs ranged from 20 to 25 nt with median at 22 nt.

### Tissue/breed specific miRNAs

**Fig. 5:**
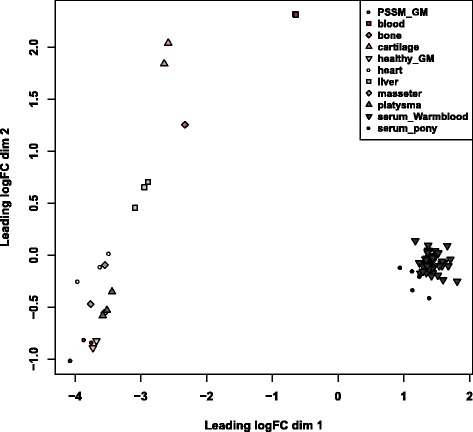
Multi-dimensional scaling plot of the RNA samples sequenced. The distances correspond to leading average (root-mean-square) of the largest absolute log-fold-changes between each pair of samples [26]

MiRNAs expression patterns differ for specific tissues differentiation states and health status. Multi-dimensional scaling MDS showed that samples clustered according to the tissue (Fig. 5), rather than by individual, thus prefiguring a relevant scenario to draw tissue-specific miRNA expression profiles [26].

**Fig. 6:**
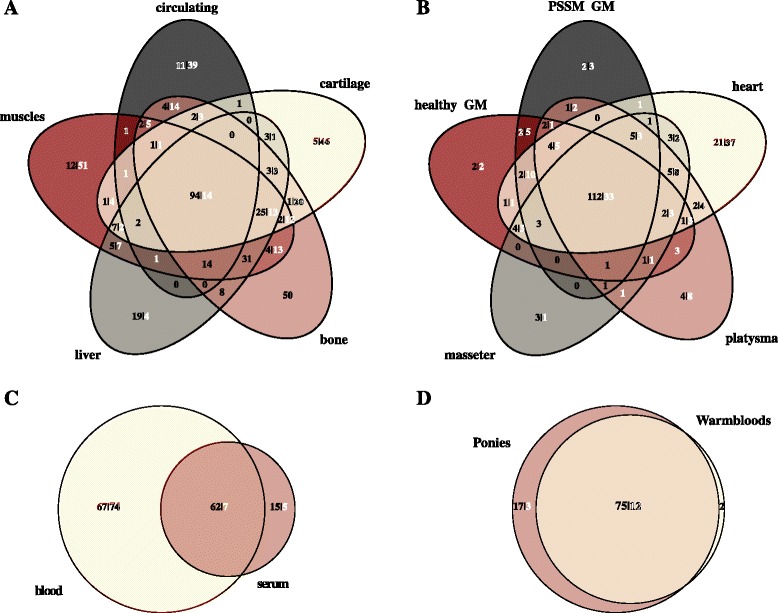
Tissue and serum breed-specific mature miRNA. The venn diagrams show the number of known mature miRNAs (black) and novel miRNAs (white) moderately or highly expressed (>10 cpm in 90 % group samples) in each condition: **a** tissue; **b** muscles; **c** circulating components; **d** breed (serum)

MiRNAs expressed at moderate to high level (≥10 cpm) in 90 % of libraries from the same tissue or breed (serum samples) were considered as expressed in the particular group (tissue/breed). From here on, we will refer to miRNAs expressed in only one group as tissue/breed specific miRNAs. Subsequently, we further explored miRNAs exhibiting differential expression between all muscle tissues together, blood and serum together, liver, bone and cartilage. The 562 miRNAs (317 novel mature miRNAs) expressed at > 10 cpm on average in at least one tissue presented different expression distribution: almost half of the miRNAs were expressed only in one tissue (42 %; 44 % novel), 4 % (liver) to 11 % (muscles) were expressed in a single tissue, and about 19 % in all the tissues: muscles, liver, cartilage, bone, and circulating (serum or blood) (Fig. 6a). The number of tissue-specific miRNAs discovered was similar and ranged from 23 (liver) to 63 (muscles) as shown in Fig. 6a.

Among muscles, the heart muscle had the highest number of specific miRNAs (*n* = 58) (Fig. 6b). Only 108 miRNAs were expressed in all the tissues at the level of > 10 cpm on average and among those only 14 were novel miRNAs. The group specific miRNAs expressed at >100 cpm on average are listed in Table 1. As depicted in the Fig. 5, the MDS plot revealed miRNA expression dissimilarity between healthy GM and PSSM GM. More precisely, we identified five miRNAs expressed at the level > 10 cpm solely in PSSM GM muscle: eca-miR-144, eca-miR-20b, ecaub_novel-miR-472, ecaub_novel-miR-568, and ecaub_novel-miR-892. The miR-144 was also expressed at >10 cpm level in bone and eca-miR-20b in bone and liver (Additional file 3: Table S2). However, none of the miRNAs was significantly up- or down-regulated in PSSM compared to healthy GM even at FDR < 0.1.

**Table 1:** Tissue-specific miRNAs and their mean expression

Tissue	Mature miRNA	Mean expression [cpm]
Cartilage	ecaub_novel-miR-174	127.47
Cartilage	ecaub_novel-miR-27	117.53
Cartilage	ecaub_novel-miR-634	106.9
Liver	eca-miR-193	254.72
Serum Pony	eca-miR-483	247.97

The table presents tissue-specific miRNAs among tissues evaluated in this study with mean expression above 100 cpm

Close to one third (*n* = 69; 33 %) of the miRNAs expressed in blood were also expressed in serum, whereas 20 miRNAs could be detected only in serum (Fig. 6c). Most of these serum-specific miRNAs were expressed in both breeds (Warmbloods and ponies), although we identified two miRNAs expressed solely in Warmblood (eca-let-7f, eca-miR-361-5p) and 20 other solely in ponies (Fig. 6d). Interestingly, among these serum-specific miRNAs expressed solely in ponies was eca-miR-483 that was expressed at >100 cpm on average (Table 1, Additional file 3: Table S2).

### Differentially expressed serum miRNAs

**Fig. 7:**
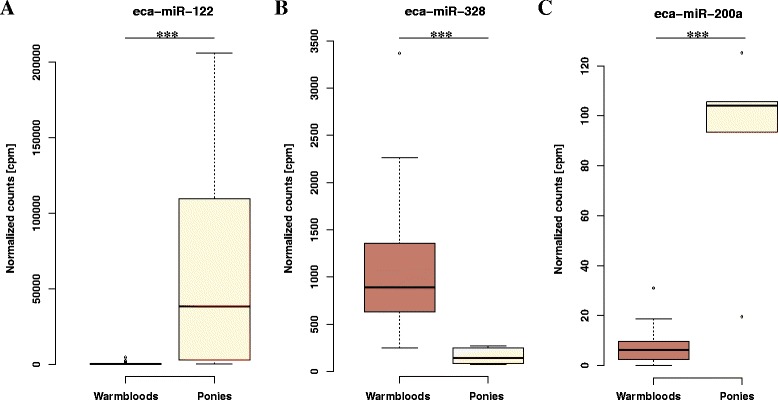
Significantly differentially expressed miRNA in the serum of ponies. Normalized expression levels of the most up- (**a**) and down-regulated (**b**) miRNA in Pony compared to Warmblood serum as well as eca-miR-200a (**c**) that is downregulated by HMGA2

MiRNAs that showed strong specificity between *Equus caballus* subspecies were determined by comparing the expression between horse and pony serum. Age and sex did not have effect on the miRNA expression profile, with no significantly differentially expressed miRNAs (DEmiRs), apart from one miRNA (eca-miR-8908j) that passed the lower significance threshold usually suggested for DEmiRs (upregulated in males with log 2 fold change (log2FC) = 4.23; FDR = 0.013). The absorbance at 414 nm had a significant effect on the expression of eca-miR-25 (FDR = 4.15e-4) and eca-miR-486-5p (FDR = 0.066) that was previously reported as haemolysis dependant [27]. We identified 50 differentially expressed (DE) miRNAs (42 known and 9 novel) between pony and Warmblood serum at a stringent significance threshold FDR < 0.001. More than half of them (*n* = 35; 67 %) were upregulated in ponies (range log2FC from 1.58 to 7.05), including 3 novel miRNAs (Additional file 5: Table S4). The remaining 16 miRNAs were down-regulated (range log2FC from −1.31 to −6.04), including 6 novel miRNAs (Additional file 5: Table S4). The most significant up- (eca-miR-122) and down-regulated (eca-miR-328) miRNAs in ponies related to Warmblood are shown on Fig. 7a-b.

We next selected a total of 177 target-genes of eca-miR-122 using TargetScan [28] and performed gene set enrichment analysis with GeneCodis [29] to assess their biological role. The only significant Kyoto Encyclopedia of Genes and Genomes (KEGG) pathway [30] was glycolysis/gluconeogenesis (KEGG: 00010, corrected hypergeometric *p*-value, Hyp* = 0.03), which encompassed four target genes: pyruvate kinase, muscle (*PKM2*), glucose-6-phosphatase, catalytic subunit (*G6PC*), dihydrolipoamide S-acetyltransferase (*DLAT*), and aldolase A, fructose-bisphosphate (*ALDOA*).

The downregulated eca-miR-328 had 204 potential target genes reported by TargetScan and these genes significantly enriched five KEGG pathways, from which the most significant was the regulation of actin cytoskeleton (KEGG: 04810, Hyp* = 0.01) with seven genes: fibroblast growth factor 1 (*FGF1*) and 11 (*FGF11*), p21 protein (Cdc42/Rac)-activated kinase 6 (*PAK6*), integrin alpha 5 (fibronectin receptor, alpha polypeptide) (*ITGA5*), v-rck sarcoma virus CT10 oncogene homolog (avian) (*CRK*), WAS protein family member 2 (*WASF2*), and FYVE, RhoGEF and PH domain containing 1 (*FGD1*). The other significant pathways were: pathways in cancer (KEGG: 05200; Hyp* = 0.02); adherens junction (KEGG: 04520, Hyp* = 0.02); renal cell carcinoma (KEGG: 05211; Hyp* = 0.03); and Wnt signalling pathway (KEGG: 04310; Hyp* = 0.04).

## Discussion

Despite high sequence conservation much less is known about equine miRNome compared to human or murine. Therefore, in this study we examined the expression of known and novel miRNAs in a comprehensive list of nine horse tissues using 71 small RNA sequencing libraries derived from 83 horses. The identification of novel miRNAs is limited by the fact that huge fraction of small RNA-seq reads is filtered out due to multiple mapping to the reference genome (n > 5 hits). These short sequences with secondary structure (hairpin) are often overlapping repeat regions [31].

As a gold standard has not been established in the field of miRNA discovery, we decided to use two reportedly well performing tools and combine the results. While 1,174 miRNAs were identified in our study by miRDeep2, only four were identified by the more stringent miRdentify. The miRdentify does not use information on other species miRNA annotations and usually results in lower number of miRNAs predicted [17]. Small overlap between those two tools is known and the stringent ten parameters of miRdentify discards 50 % of human annotated miRNAs [17]. Whereas all the four miRNAs identified by miRdentify partially overlapped the known or Ensembl predicted miRNA genes, novel miRNAs identified by miRDeep2 often spanned unannotated regions of the horse genome.

After removing lowly expressed miRNAs, our dataset consisted of 683 novel miRNAs (Fig. 1), from which 49 % were transcribed from the sense strand, accordingly to known equine miRNAs that are annotated equally on both strands (miRBase, release 21). The highest number of novel miRNAs was identified on the largest chromosome one, but also 40 novel miRNAs were identified on unplaced chromosomes (Fig. 4). The unplaced chromosomes are often neglected, as it is the least annotated region of the horse genome. A future EquCab3 reference genome is believed to decrease the number of unplaced scaffolds and improve the quality of the reference genome. Interestingly, we identified a putative novel miRNA encoded by the mitochondrial genome that has been previously described in horse [32], however, the mitomiR was identified only in one heart sample and was excluded from further analysis.

Our study aimed to identify tissue-/serum breed-specific miRNAs and our definition of such miRNAs presumed that specific miRNA can be expressed at low level (<10 cpm) in other tissues but it has to be expressed at least at the level of 10 cpm in 90 % of the samples of a particular tissue/breed. This approach differs from other studies that were based on quantitative reverse transcription PCR (RT-qPCR) or microarray data, where “yes/no” approach was taken. However, we hypothesize that since miRNAs can be exported and transferred between the cells, they might appear in low amounts in other tissues that they do not originate from and where they are not directed to. Moreover, it has been shown that only 200 miRNAs were expressed in moderate to high levels (>10 cpm) in human brain and most likely the moderately/highly expressed miRNA play more important role in gene expression regulation [23–25].

With our approach, we identified miRNAs that were expressed in a tissue- and breed-specific manner (Fig. 6a). We identified a group of 108 miRNAs that were universally expressed at >10 cpm in 90 % of libraries across all the tissues studied (Fig. 6a). Such a feature identifies this group of miRNAs as a candidate universal reference to normalize miRNA expression in normal horse tissues. The expression pattern and tissues distribution of these 108 miRNAs suggest that they might be associated with biological fundamental functions in the cell, such as metabolism. We identified 40 miRNAs that were expressed in specific tissues with minimal or no expression in other tissues we examined, suggesting more local function of these miRNAs that are not exported outside the cell. For example, eca-miR-1 and eca-miR-133a were highly expressed primarily in muscles and we observed very little or no expression of these miRNAs in other tissues. A total of eight serum-specific miRNAs (eca-miR-1307, eca-miR-1379, eca-miR-7177b, eca-miR-9021, ecaub_novel-miR-1145, ecaub_novel-miR-262, ecaub_novel-miR-79, ecaub_novel-miR-932) were expressed in a tissue-specific manner, although they may derive from other tissues not analysed in the present study and therefore have to be treated with caution.

The heart tissue had the most specific miRNAs among all the muscles (Fig. 6b). The cardiac muscle is highly specialized tissue and is a different type compared to other skeletal muscles evaluated in this study and therefore more specific miRNAs were expected to be expressed in the heart tissue [33]. Moreover, many miRNAs have been already implicated in the cardiac development and diseases [34, 35]. For such as limited number of libraries per group examined, several miRNAs that were previously considered as “tissue-specific” in humans were not detected in our data. For instance, miR-208, miR-302 (a-d), miR-367 were not detected (at >10 cpm on average) in the heart tissue; miR-134 and miR-208a were not detected in skeletal muscles; miR-483 in liver; or miR-483 and miR-377 in bone [36].

We also noticed that only less than half of the miRNAs expressed in blood (33 %) were common with serum miRNAs, suggesting that remaining 67 % of miRNAs is expressed in blood cells and are not exported to serum (Fig. 6c). However, we need to take into account that the serum miRNA set was derived from different horse breed and some additional breed specific differences may apply as we shown for serum samples in Fig. 6d.

Our study also provided an opportunity to re-visit and confirm tissue-specific miRNAs previously reported in horses. Interestingly only five novel cartilage/bone miRNAs reported in [10] have been also identified in this study, however, the mature miRNAs reported here were slightly longer. Interestingly two of them were not expressed (at >10 cpm level) in either cartilage or bone, but in blood (Additional file 3: Table S2 and Additional file 4: Table S3). From five most abundantly expressed novel putative miRNA reported in equine plasma by Lee et al. [37], only one miRNA was identified in our study in bone and blood tissues with a mature sequenced shorter by three nucleotides from 5’ end. From the 329 novel miRNAs reported by Kim et al. [9] only 11 had exactly the same mature sequence as novel miRNAs reported here. From the 896 putative novel miRNAs included in the microarrays used by Mach et al. [32] only two had exactly the same mature sequence and further 24 had few nucleotides shorter mature sequence compared to the miRNAs identified here (Additional file 4: Table S3). One of the reasons of such discrepancy is the different method used for putative novel miRNA identification in all of those studies. Moreover, a phenomenon of multiple isoforms (isomiRs) derived from the same precursor is known, where a pre-mature or mature sequence of a miRNA is modified by an addition, edition, or subtraction of nucleotides [38, 39]. For single miRNA, even a total of about 7000 isomiRs, including combinations, can exist and consequently sRNA-seq can show/generate a large part of these isomiRs [40].

Consistent with literature findings, miR-206 and miR-133b were characterized solely in muscle, where they promote differentiation or proliferation of myoblasts [41]. In addition, non-muscle-specific miRNA involved in myogenesis (e.g. miR-24, miR-181 and mir-214) were broadly expressed [41]. Nonetheless, from the 166 tissue-specific known equine miRNAs reported in liver, colon or muscles, a total of 132 were expressed in our study and 105 of them were expressed at >10 cpm on average in at least one group. Interestingly, 21 miRNAs reported as tissue-specific in Kim et al. [9] were also expressed in all groups studied here, at > 10 cpm on average. From the 31 liver-specific miRNAs reported by Kim et al. [9], only one was supported by our study with our definition of tissue specificity (eca-miR-135a). On contrary, four of liver-specific miRNAs reported here were reported in Kim et al. [9] as muscle (eca-miR-299, eca-miR-32, eca-miR-656) or colon-specific miRNAs (eca-miR-429). It was not clear to us, how the authors determined the tissue specificity and there is a clear need to establish a common standard for future reference.

Several recent studies have supported the possibilities for the use of miRNA signatures as biomarkers for the detection of cancer, pregnancy and other diseases such as myopathies in humans and murine models. In our study, the expression of miRNAs specific for PSSM-GM-were not statistically differentially expressed relative to healthy animals, which most likely was due to low number of samples and/or general low expression of these specific miRNAs.

Because a significant genetic diversity (~10 %) and structure between horse breeds has been described [42], it is tempting to speculate that this genetic variability may result in different miRNA synthesis and expression that may contribute to phenotypic variation between Warmblood and ponies. In fact, we identified 50 DEmiRs between Warmblood and pony serum samples (Additional file 5: Table S4). The most significantly DEmiR was eca-miR-122, which was highly up-regulated in ponies compared to Warmbloods (Fig. 7a). This miRNA, which is highly expressed in liver [43, 44], was also expressed at the highest level in the liver samples (mean = 43,681 cpm). The human miR-122 is involved in glucose and lipid metabolism [45, 46] and it has been proposed as a therapeutic target for metabolic diseases [46]. Ponies are known to be among breeds more prone to develop equine metabolic syndrome (EMS) [47] and therefore miR-122 may be of particular interest for unravelling the molecular mechanism of this disease. Moreover, the pony serum specific miR-483, also significantly upregulated in ponies (FDR = 4.15e-8), has been shown to be upregulated in patients with adrenocortical cancer [48, 49]. This may suggest that eca-miR-483 might potentially increase the predisposition to metabolic diseases in ponies.

Moreover, it was reported that high mobility group AT-hook 2 gene (*HMGA2*) carries a SNP associated with height in Shetland Pony [50]. HMGA2 also down-regulates the expression of miR-200b [51]. Both miR-200a and miR-200b were significantly upregulated in pony serum in our study with log2FC >3.5 (Fig. 7c, Additional file 3: Table S2). Therefore, we can speculate that impaired HMGA2 has decreased capability to inhibit the miR-200 expression in ponies.

To confirm the breed specificity of the serum miRNAs more serum samples from various horse breeds are necessary to analyse in terms of their miRNA profile in future. However, this study shows clear differences in miRNA profiles between ponies and Warmbloods.

## Conclusions

Our sRNA-seq data and analyses of expression patterns in 9 different tissues presented a global view of tissue distribution of known and novel miRNAs and the relation to their chromosomal locations. We identified 683 novel equine miRNAs expressed in seven solid tissues, blood and serum. Additionally, our approach evidenced that such data supported identification of specific miRNAs as markers of functions related to breeds or disease tissues. For instance, we showed an increased expression of circulating serum miR-122 and miR-200 in ponies together with the predicted miRNA target genes that are required in the control of energy metabolism. The increased expression of eca-miR-483 in ponies relative to Warmblood suggested an increased predisposition to metabolic diseases in ponies. Lastly, we were also able to identify miRNAs not exported to the serum, which most likely have local function.

## Methods

### Samples, RNA extraction and RNA-seq

The SOLiD libraries were prepared from g*luteus medius*, *platysma*, *masseter*, heart (exterior cardiac muscle), cartilage, liver, bone, and blood samples derived from a total of 35 horses of different age and breed (collected at the slaughterhouse). Two SOLID libraries were prepared from two blood samples collected from one endurance horse of Arabian breed aged of 10 years-old before and after 2 h of training exercise at canter, respectively. Nine SOLiD libraries were prepared from cartilage and subchondral bone explants collected in the middle trochlea of healthy stifle joint from four 6 months old Anglo-Arabian horses [10]. Two cartilage libraries were prepared from the same set of three horses, however, derived from a different sample collection. The bone library also consist of pooled RNA samples derived from three horses. The SOLiD libraries prepared from g*luteus medius*, *platysma*, *masseter*, heart and liver were derived from 30 horses (12.5 ± 5.6 yo), pooled by three animals of different breeds 15 Cob Normand, 8 French trotter, 6 Selle Français, and 1 Trait du Nord. More details on the samples can be found in Additional file 1: Table S1. Different types of muscles were chosen to better understand their roles in the regulation of physiological processes and metabolism in skeletal muscles. While *masseter* contains a type I slow oxidative fiber muscle, *platysma* is enriched in type IIX fast glycolytic fiber muscle and *gluteus medius* predominantly contains type IIB fast glycolytic fiber. On the other hand, heart is highly resistant to fatigue because its high content of mitochondria, which enables continuous aerobic respiration via oxidative phosphorylation. After collection in the slaughterhouse, 60 mg of tissue samples were cut in small pieces and lysed in 1 ml of Trizol reagent (Invitrogen, Cergy Pontoise, France) with ceramic beads (Bertin technologies, St Quentin en Yvelines, France). Epiphyseal cartilage and subchondral bone were obtained from healthy stifle joint (macroscopical examination at necropsy). Whole blood was sampled from one Arabian horse before the 160 km ride and then again afterwards.

The mirVana™ miRNA Isolation Kit (Ambion, Life Technologies) was used to extract in parallel Total RNA and small RNA (smRNA) from tissues following the manufacturer’s protocol, whereas total blood RNA was isolated using a PAXgene Blood RNA Kit (Qiagen), according to the manufacturer’s instructions. Total RNA was purified using RNeasy Mini Kit (Qiagen, Courtaboeuf, France) according to the manufacturer's recommendations. Residual genomic DNA was removed using DNase digestion with RNase-free DNase I Amp Grade (Invitrogen, Cergy Pontoise, France) following the recommended protocol. RNA concentration was measured by using a NanoDrop spectrophotometer (NanoDrop Technologies, Wilmington, USA), and the RNA integrity value (RIN) was assessed by using a 2100 Bioanalyzer (Agilent Technologies Inc., Santa-Clara, USA). All samples had an RNA integrity number (RIN) > 8. For further details on SOLiD samples, preparation and sequencing, see [10, 32].

A total of 22 libraries were prepared: five for *gluteus medius* muscle; three for heart, liver, *masseter*, and *platysma* muscle; two for cartilage and blood, and one for bone. Among the five libraries prepared from *gluteus medius* muscle, two were prepared from healthy muscle, three from horses affected by polysaccharide storage myopathy, type I, PSSM. The PSSM-affected horses were diagnosed based on *GYS2* genotype and histological examination of glycogen digestion as described in [52, 53]. Within each library, three different horses were pooled to decrease the effect of the individual variability, except for the blood samples where each library comes from the same animal (Additional file 1: Table S1) but the samples come from different physiological moments (before and after exercise [32]. Approximately 200 ng of mRNA extracted from the different tissues and blood were used for library construction following the protocol for the SOLiD Total RNA-Seq Kit (Applied Biosystems, Life Technologies). Libraries were sequenced with 50 cycles on 5500XL Series Genetic Analysis System at the Metaquant core facility (INRA, Jouy-en-Josas, France).

The serum samples were collected from 31 Swiss and 13 other European Warmblood horses (22 males and 22 females, aged 6–32 years, average = 18 years), and four Shetland and one Welsh pony (three males and two females, aged 5–19 years, average = 12 years). Briefly, blood was collected from jugular vein into sodium-heparin-containing vacutainers [54]. Within 1–6 h serum was separated from cells via centrifugation 1800 g for 20 min. Serum was then transferred to new tubes and stored in −80 °C until RNA extraction (up to 4 years). In order to verify the level of haemolysis in serum samples we used VersaMax ELISA Microplate Reader (Molecular Devices) and SoftMax Pro software (version 3.1.2). The absorbance in 200 μl of serum was measured at 414 nm wavelength, which is the length of maximum absorbance for haemoglobin [55, 56]. The RNA was extracted from 2 ml of serum using miRNeasy serum/plasma kit as described in [20]. The concentration of RNA extracted was measured with Qubit RNA High Sensitivity kit and ranged from 2 to 428 ng/μl (20 ng/μl on average). Twelve samples were randomly chosen for the small RNA length distribution assay with Bioanalyzer 2100 small RNA chip. The miRNA in small RNA content of these 12 samples ranged from 69 to 89 % (81 % on average). The RNA was successfully converted into single-end libraries with NEBNext Multiplex Small RNA Library Prep Set for Illumina (New England BioLabs) and sequenced on an Illumina HiSeq 2500 with 50 sequencing cycles.

### Data pre-processing

The raw sequencing reads from each library were check for quality with FASTQC v. 0.11.2 [57]. The sequencing data was processed in the following manner: 1) multiple adapter sequences were trimmed with cutadapt (v. 1.8) with the following options: -c -a ‘adapter sequence’ -m 18 -M 30 --trim-n -q 15,10 [58] for SOLiD data; or --trim-n -b ‘sample specific adapter sequence’ -m 15 for HiSeq data; 2) trimmed reads were mapped to the *Equus caballus* reference genome EquCab2 v2.74 [59] with BowTie (v. 0.12.9) [21] with the following options: -n 1 -l 8 -a -m 5 --best --strata (for colour-space SOLiD reads additional options were used: -c --col-keepends). We required small RNAs to map with one base mismatches as recommended in [60]. Reads mapping more than five times to the genome were discarded. The mapping quality and read distribution was analysed with RSeQC (v. 2.6.1) [61]. We inspected the read distribution among: i) known equine genes without miRNA genes (Ensembl, release 81); ii) known equine miRNA genes; iii) repeating elements annotated in horse genome (based on RepeatMasker [62, 63]). Known equine and human miRNA mature and hairpin sequences were downloaded from miRBase, release 21 [64].

The pre-processed mapped reads in BAM format were deposited at the European Nucleotide Archive (http://www.ebi.ac.uk/ena/data/view/PRJEB14485).

### miRNA discovery

We used two different tools for miRNA discovery: (i) miRDeep2 (v. 0.0.7) [15, 16] and (ii) miRdentify (v. 1.0), which is high stringency predictor [17]. The miRNA identification analysis was performed independently in each sample to allow for authentic detection of miRNAs. The high-quality sequencing reads were used as input data. Briefly, the miRDeep2 algorithm excises the sRNA sequence and computes their secondary RNA structure with RNAfold [65] to predict miRNA precursors that are later scored for their likelihood [15]. A total miRDeep2 score was designated based on an algorithm incorporating the statistics of read positions, read frequencies within stem-loops, and posterior probability that the stem-loop was derived from an authentic miRNA. To prevent false positive detection of miRNA stem-loops, the signal-to-noise ratios estimated over 100 rounds of independent permutations were calculated for different miRDeep log-odds score cut-offs from −10 to 10. For a prediction of potential horse specific RNAs, a criterion of miRDeep score of 1 was adopted as a cut-off point. Therefore, all precursors with total miRDeep scores above the cut-off point were considered as putative miRNAs. The miRDeep2 tool allows for guided search for novel miRNA with an optional set of miRNAs from a related species. As the miRNA annotations in horse genome are relatively poor, we used the most comprehensive set of human miRNAs as guidance. Known equine miRNAs were obtained from the miRBase database, release 21 [13]. In addition, human known miRNAs from miRBase were used as “related species” set of miRNAs. The putative miRNA identified by the miRDeep2 program or miRdentify that did not show a significant match to miRBase were regarded as novel miRNAs. The miRdentify tool applies 10 additional parameters: heterogeneity at both 5’ and 3’ ends (low heterogeneity is expected), 5’ and 3’ arm overhang (two nucleotide 3’ overhangs at both ends), duplex and flanking sequence minimal free energy (MFE; lower MFE is desirable), nucleotide and structural entropy (higher degree of nucleotide entropy and lower structural complexity of the hairpin are desirable), tailing (high level of tailing with adenosine or uridine); and multimap factors (reads with more than four genomic hits are discarded) [17].

The miRNAs were identified for each sample separately. MiRNA that differed only by additional two nucleotides at the start/end position were considered as a single miRNA and the miRNA that was identified in the highest number of samples or had a higher miRDeep2 score was chosen as a representative and most likely true miRNA. Novel miRNA identified in one sample only were filtered out.

We then combined the novel and known miRNA and the miRNA expression for each sample was determined with miRDeep2 quantifier tool [16]. Mature miRNAs expressed at < 1 cpm in 90 % of samples were filtered out. Then, miRNAs expressed at ≥ 10 cpm in 90 % of group samples were considered as expressed in the group analysed (tissue/breed). To analyse whether miRNAs were ubiquitously expressed among the targeted tissues, a Venn diagram was plotted by using “venn.diagram” function from VennDiagram R package.

### Differential expression analysis of serum samples

The MDS plots, which measure the differences between samples and project it into two dimensions, were through the “MDSplot” function of edgeR package. The estimated raw counts of each miRNA from miRDeep2 output were used for the differential expression analysis using edgeR package. To avoid inclusion of miRNAs containing too few reads, as recommended by authors of the package, only mature miRNAs with the expression > 10 cpm in 90 % of the samples were kept for the analysis. In order to discover biologically important changes in expression, the TMM normalization method [66] of edgeR package was applied (“calcNormFactors” function) and the dispersions were calculated based on the quantile-adjusted conditional maximum likelihood (“estimateDisp” function). The differentially expressed (DE) genes were detected by applying an exact test [67]. The edgeR generated a fold change for each miRNA, *P*-values and the associated Benjamin-Hochberg false discovery rate (FDR) values. MiRNAs showing a FDR < 0.001 were considered as DE.

### Biological interpretation of the analysis

The potential targets of tissue and serum breed-specific miRNAs were identified using TargetScan (release 6.2) [28] and subjected for gene set enrichment analysis with GeneCodis [29] to infer the main biological functions associated the different tissues. We used KEGG pathways annotated for human, as equine annotations were not available.

## Additional files

Additional file 1: Table S1. RNA sample description. An Excel table with details on RNA samples used for library preparation. For each library horse ID, tissue, breed, age, sex, and absorbance at 414 nm (only serum samples) are given. (XLSX 17 kb)
Additional file 2: Figure S1. miRNA gene body coverage. The read coverage of known miRNAs genes per library. On the Y axis coverage measured by Pearson’s skewness coefficients. All transcripts were scaled into 100 nt and the length is denoted on the X axis (RSeQC v.2.6.1). (PDF 33 kb)
Additional file 3: Table S2. Known and novel miRNAs expression values. An Excel table with normalized count per million values (cpm) for each of the known and novel miRNAs expressed at > 1 cpm in 90 % of samples. (XLSX 519 kb)
Additional file 4: Table S3. Novel miRNA characteristics. An Excel table with novel miRNAs identified in this study. Mature miRNA sequences, genomic coordinates of the hairpins, strand and miRDeep2 score are given. (XLSX 85 kb)
Additional file 5: Table S4. Differentially expressed miRNAs in pony serum compare to Warmbloods. An Excel table with log 2 fold changes and false discovery rates of genes significantly differentially expressed in serum derived from 4 Shetland and one Welsh pony in comparison to 44 Warmbloods. (XLSX 11 kb)

## Abbreviations

CDS: Coding DNA sequences
cpm: Count per million
FDR: False discovery rate
GM: *Gluteus medius*
Hyp*: Corrected hypergeometric *p*-value
KEGG: Kyoto Encyclopedia of Genes and Genomes
MDS: Multi-dimensional scaling
miRNA: Micro RNA
PSSM: Polysaccharide storage myopathy type I
rRNA: Ribosomal RNA
RT-qPCR: Quantitative reverse transcription polymerase chain reaction
sRNA-seq: Small RNA sequencing
TMM: Trimmed means of M values
tRNA: Transfer RNA
UTR: Untranslated region
