# Supplementary figures and images for: Novel equine tissue miRNAs and breed-related miRNA expressed in serum
Source: BMC Genomics. 2016 Oct 26;17:831. doi: 10.1186/s12864-016-3168-2 (PMC5080802; doi:10.1186/s12864-016-3168-2)

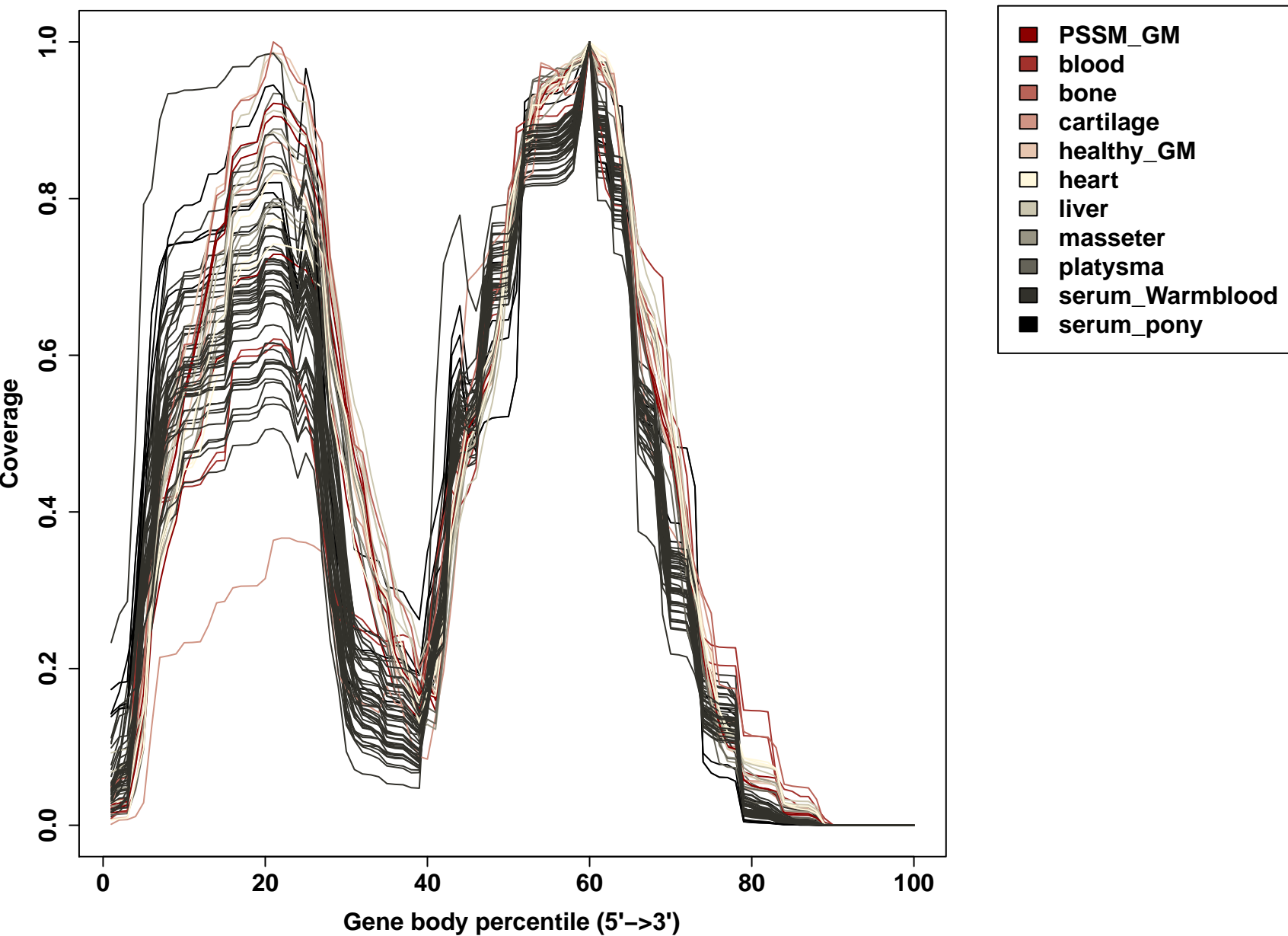

Supplement: Additional file 2: Figure S1. — miRNA gene body coverage. The read coverage of known miRNAs genes per library. On the Y axis coverage measured by Pearson’s skewness coefficients. All transcripts were scaled into 100 nt and the length is denoted on the X axis (RSeQC v.2.6.1). (PDF 33 kb) [file 12864_2016_3168_MOESM2_ESM.pdf]
